# Supplementary material for: Volatile Compounds in Fruit Peels as Novel Biomarkers for the Identification of Four Citrus Species
Source: Molecules. 2019 Dec 12;24(24):4550. doi: 10.3390/molecules24244550 (PMC6943597; doi:10.3390/molecules24244550)
Supplement: Supplementary file 1 [file molecules-24-04550-s001.pdf]

Table S1 Materials and groups were used in this study.

Citrus germplasms	Species name	Group	Wild or Cultivar
Huanongbendizao tangerine		LSM	Cultivar (C)
Guinongbendizao tangerine		LSM	C
Guizhouwuhezhuju tangerine		LSM	C
Red Tangerine		LSM	Wild (W)
Huangyanbendizao tangerine		LSM	C
Nanfengmiju tangerine		LSM	C
Nianju tangerine		LSM	C
Nieduyedonggan		LSM	W
E-gan No.1 Ponkan tangerine		LSM	C
Huapiju tangerine		LSM	C
Wuhe Ponkan tangerine		LSM	C
India sour Tangerine	Loose-skin mandarin (*Citrus reticulate*)	LSM	W
Wulong Sour Tangerine		LSM	W
Cupigoushigan tangerine		LSM	W
Dakengyeju tangerine		LSM	W
Daoxianyeju tangerine		LSM	W
Jiangyongyeju tangerine		LSM	W
Mangshanju tangerine		LSM	W
Xipigoushigan tangerine		LSM	W
Damaliu tangerine		LSM	W
Yaoxianggan tangerine		LSM	W
Miyagawa Wase		LSM	C
Guangmingzao		LSM	C
Guoqing No.4		LSM	C

Changyang Kamei		LSM	C
Dapu		LSM	C
Inaba Wase		LSM	C
Guoqing No.1		LSM	C
Nichinan No.1 Wase		LSM	C
Caffion clementine	Clementine mandarin (*C. reticulate* × *C. sinensis*)	CCL	C
De nules clementine		CCL	C
Hernadinal clementine		CCL	C
Marisal clementine		CCL	C
Monreal clementine		CCL	C
Nour clementine		CCL	C
Orograde clementine		CCL	C
Oroval clementine		CCL	C
Pons clementine		CCL	C
Rrecoce clementine		CCL	C
Rubino clementine		CCL	C
Sidiaissa1 clementine		CCL	C
Spinoso.V,C,R clementine		CCL	C
SRA63 clementine		CCL	C
SRA92 clementine		CCL	C
Tomatera clementine		CCL	C
Anliucheng	Sweet orange (*C. sinensis*)	SW	C
Qianyang Wuhe Dahong		SW	C
Hamlin Sweet Orange		SW	C
Red Anliucheng		SW	C
Meishan Wuhejincheng		SW	C

Taoye sweet orange		SW	C
Xuegan		SW	C
Qingpi		SW	C
Carter Valencia orange		SW	C
Frost Valencia orange		SW	C
Crame Navel Orange		SW	C
Palmer Navel Orange		SW	C
Red grand Navel Orange		SW	C
Roberson Navel Orange		SW	C
Smithearly Navel Orange		SW	C
Fukumoto Navel Orange		SW	C
Red Flesh Navel Orange		SW	C
Washington Naval Orange		SW	C
Dream Navel Orange		SW	C
Nice navel Orange		SW	C
Newhall Navel Orange		SW	C
Seike Navel Orange		SW	C
Xiatian Navel Orange		SW	C
Zaohong Navel Orange		SW	C
Huanonghongyou Pomelo		P	C
Taiyou Pomelo		P	C
Acidless Pomelo		P	C
Fenghuangyou Pomelo	Pomelo (*C. grandis*)	P	C
Fenghuangyou Pomelo		P	C
Juanpi Pomelo		P	C
Ni 800 Pomelo		P	W

Kuigan Pomelo		P	W
Lime		Lem	W
Eureka Lemon		Lem	C
Volkamer lemon	Lemon (*C.limon*)	Lem	C
Limonia		Lem	W
Red Limonia		Lem	W

Note LSM: loose-skin mandarin; CCL: clementine mandarin; SW: sweet orange; P: pomelo; Lem: lemon.

Table S2: Volatile compounds were used to PLS-DA in four citrus species.

Abb.	compounds	ID	Abb.	compounds	ID
C1	α-pinene	S	C46	γ-elemene	T
C2	sabinene	S	C47	*trans*-α-bergamotene	T
C3	β-pinene	S	C48	α-guaiene	T
C4	β-myrcene	S	C49	germacrene D	T
C5	α-phellandrene	S	C50	elixene	T
C6	α-terpinene	S	C51	α-selinene	T
C7	d-limonene	S	C52	α-muurolene	T
C8	β-cis-ocimene	S	C53	δ-guaiene	T
C9	*trans*-β-ocimene	S	C54	β-bisabolene	T
C10	γ-terpinene	S	C55	δ-cadinene	T
C11	terpinolene	S	C56	germacrene B	T
C12	α-thujene	T	C57	*trans*-nerolidol	S
C13	camphene	T	C58	elemol	T
C14	pseudolimonen	T	C59	germacrene D-4-ol	T
C15	*cis*-sabinene hydrate	S	C60	β-selinenol	T
C16	β-linalool	S	C61	caryophyllene oxide	S
C17	terpinen-4-ol	S	C62	2,6,10-trimethyl-2,6,9,11-dodecatetraenal	T
C18	α-terpineol	S	C63	α-sinensal	T
C19	nerol	S	C64	1-octanol	T
C20	β-citronellol	S	C65	hexanal	T
C21	*cis*-p-mentha-2,8-dien-1-ol	T	C66	(E)-2-hexenal	T
C22	citronellal	S	C67	octanal	T
C23	β-citral	S	C68	nonanal	S
C24	(E)-α-citral	S	C69	decanal	S

C25	perillal	T	C70	(Z)-2-decenal	S
C26	d-camphor	S	C71	undecanal	S
C27	(+)-carvon	S	C72	dodecanal	S
C28	(+)-p-mentha-1,8-dien-3-one	T	C73	3-hexenal	T
C29	*cis*-limonene oxide	S	C74	n-hexadecanoic acid	T
C30	*trans*-limonene oxide	S	C75	methyl palmitate	S
C31	citronellyl acetate	S	C76	butyl acetate	T
C32	nerol acetate	S	C77	acetic acid, octyl ester	T
C33	geranyl acetate	S	C78	octyl butanoate	T
C34	perillyl acetate	T	C79	methyl linoleate	T
C35	caryophyllene	S	C80	methyl oleate	T
C36	(Z)-β-farnesene	S	C81	methyl stearate	T
C37	α-caryophyllene	S	C82	undecane	T
C38	valencene	S	C83	dodecane	T
C39	α-farnesene	S	C84	tetradecane	T
C40	δ-elemene	T	C85	p-cymene	T
C41	α-cubebene	T	C86	thymol	T
C42	copaene	T	C87	methyl thymyl ether	T
C43	β-cubebene	T	C88	nootkatone	T
C44	β-elemene	T	C89	limettin	T
C45	*cis*-α-bergamotene	T			

Note T: tentatively identified compounds; S:compounds identified based on authentic standards.

Table S3: The potential biomarkers were selected in Clementine mandarin and Wild citrus germplasms.

Clementine mandarin		
Abb.	Compounds	VIP value
CM1	(Z)-β-farnesene	2.13
CM2	dodecanal	2.08
CM3	decanal	2.01
CM4	α-terpineol acetate	1.89
CM5	α-muurolene	1.83
CM6	nerol acetate	1.77
CM7	ylangene	1.76
CM8	α-sinensal	1.75
CM9	*cis*-p-mentha-2,8-dien-1-ol	1.63
CM10	γ-terpinene	1.63
CM11	2,6,10-trimethyl-2,6,9,11-dodecatetraenal	1.6
CM12	citronellal	1.56
CM13	sabinene	1.53
CM14	allo-ocimene	1.5
Wild citrus germplasms		
Abb.	Compounds	VIP value
WM1	germacrene D	2.26
WM2	*trans*-nerolidol	2.12
WM3	germacrene B	2.08
WM4	γ-elemene	1.97
WM5	β-selinenol	1.94
WM6	(+)-p-mentha-1,8-dien-3-one	1.93
WM7	citronellyl acetate	1.81

WM8	d-camphor	1.8
WM9	β-linalool	1.8
WM10	citronellal	1.76
WM11	*trans*-β-ocimene	1.71
WM12	α-pinene	1.7
WM13	δ-elemene	1.69
WM14	β-citronellol	1.67
WM15	α-thujene	1.63
WM16	germacrene D-4-ol	1.62
WM17	3-hexenal	1.62
WM18	elemol	1.57
WM19	α-terpinene	1.54
WM20	γ-terpinene	1.53

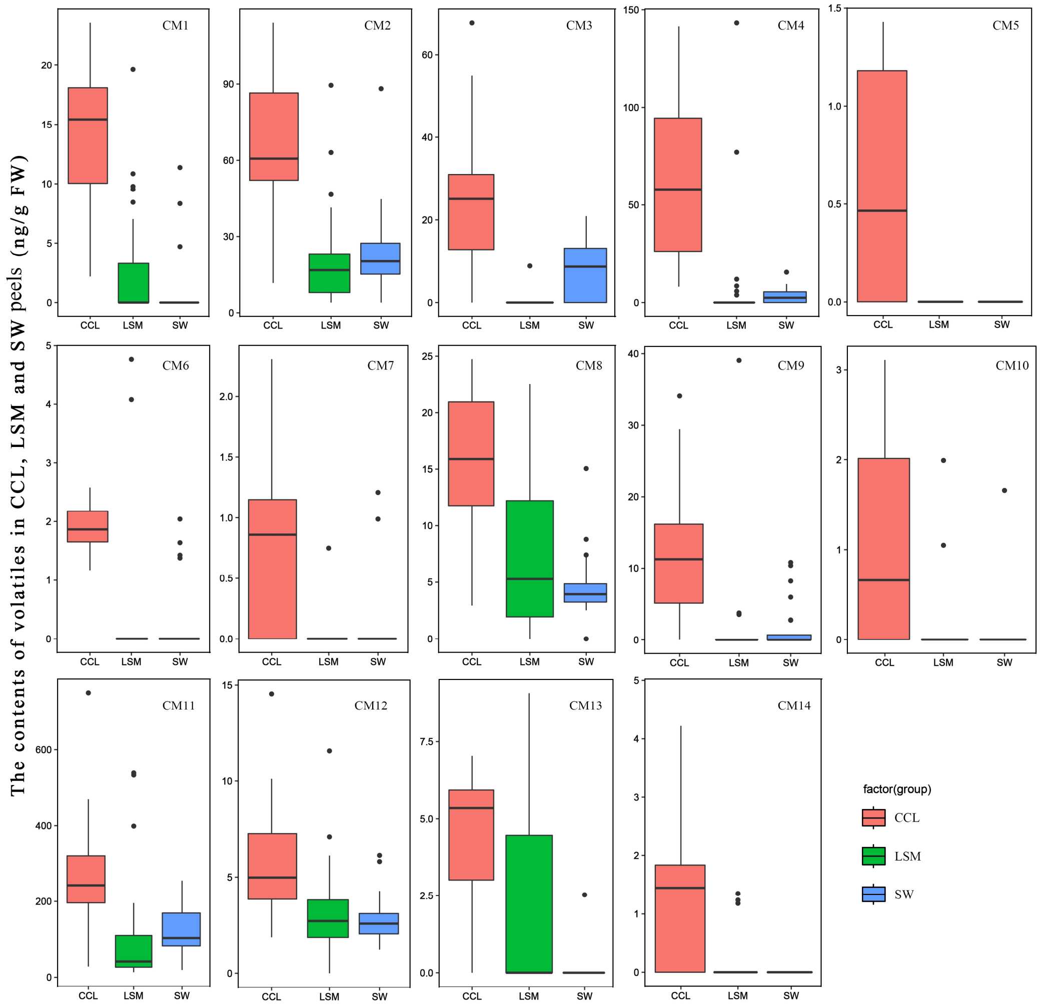

Figure 1. The contents of volatiles in CCL, LSM and SW peels. CCL: clementine mandarin; LSM: loose-skin mandarin; SW: sweet orange. The compounds were listed in the Table S3.

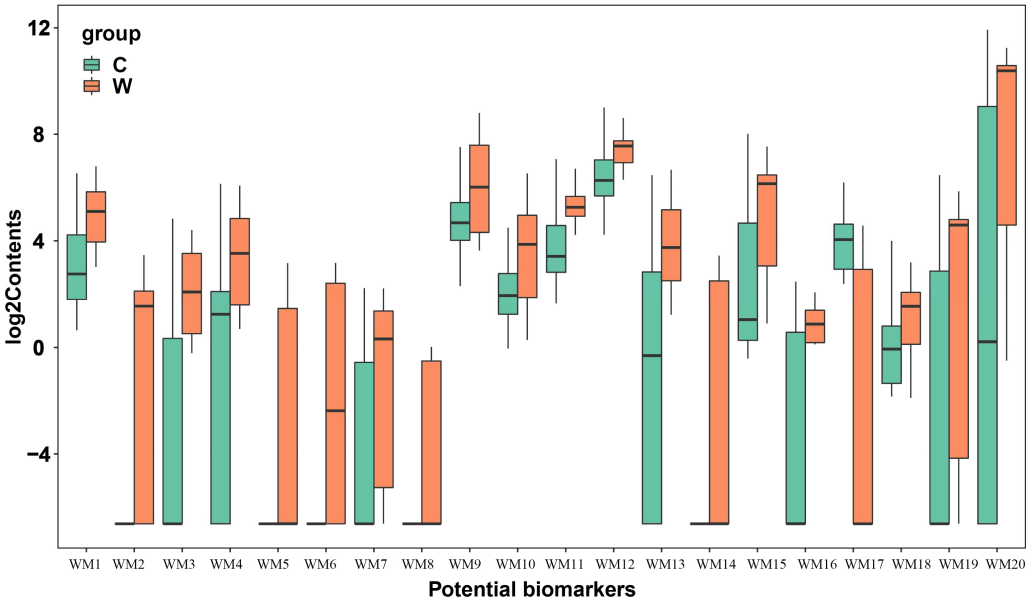

Figure S2: The Contents of potential biomarkers in wild and cultivar germplasms. The contents of volatiles were normalized by log2. The compounds were listed in the Table S3. C: Cultivar germplasms; W: Wild citrus germplasms.
